# PKM2 coordinates glycolysis with mitochondrial fusion and oxidative phosphorylation

**DOI:** 10.1007/s13238-019-0618-z

**Published:** 2019-03-18

**Authors:** Tong Li, Jinbo Han, Liangjie Jia, Xiao Hu, Liqun Chen, Yiguo Wang

**Affiliations:** MOE Key Laboratory of Bioinformatics, Tsinghua-Peking Joint Center for Life Sciences, School of Life Sciences, Tsinghua University, Beijing, 100084 China

**Keywords:** PKM2, MFN2, mTOR, glycolysis, oxidative phosphorylation

## Abstract

A change in the metabolic flux of glucose from mitochondrial oxidative phosphorylation (OXPHOS) to aerobic glycolysis is regarded as one hallmark of cancer. However, the mechanisms underlying the metabolic switch between aerobic glycolysis and OXPHOS are unclear. Here we show that the M2 isoform of pyruvate kinase (PKM2), one of the rate-limiting enzymes in glycolysis, interacts with mitofusin 2 (MFN2), a key regulator of mitochondrial fusion, to promote mitochondrial fusion and OXPHOS, and attenuate glycolysis. mTOR increases the PKM2:MFN2 interaction by phosphorylating MFN2 and thereby modulates the effect of PKM2:MFN2 on glycolysis, mitochondrial fusion and OXPHOS. Thus, an mTOR-MFN2-PKM2 signaling axis couples glycolysis and OXPHOS to modulate cancer cell growth.

**Electronic supplementary material:**

The online version of this article (doi:10.1007/s13238-019-0618-z) contains supplementary material, which is available to authorized users.

## Introduction

Normal cells catabolize glucose by mitochondrial OXPHOS to produce ATP in the presence of oxygen, whereas most cancer cells metabolize glucose by aerobic glycolysis to produce ATP and lactate, a phenomenon called the Warburg effect (Vander Heiden et al., 2009; Hanahan and Weinberg, 2011; Vander Heiden and DeBerardinis, 2017). However, the mechanisms underlying the transition from mitochondrial OXPHOS to aerobic glycolysis in cancer cells remain elusive. Previous work demonstrated that PKM2, a preferred splice isoform of pyruvate kinase in cancer cells that converts phosphoenolpyruvate (PEP) to pyruvate as the final step of glycolysis, is critical for aerobic glycolysis in cancer cells (Christofk et al., 2008a; Christofk et al., 2008b; Yang and Lu, 2015; Dayton et al., 2016).

Impaired mitochondrial dynamics and quality control are also linked to the metabolic features of cancer cells, but the molecular details of how mitochondrial dynamics affects cell metabolism are poorly understood (Gogvadze et al., 2008; Youle and van der Bliek, 2012; Liesa and Shirihai, 2013; Mishra and Chan, 2014; Chen and Chan, 2017). Mitochondria are highly dynamic organelles that constantly undergo fusion and fission of both outer and inner membranes to generate tubular or fragmented mitochondria, while the equilibrium and fidelity of mitochondrial fusion and fission maintain the electrochemical gradient for OXPHOS and determine mitochondrial quality control in response to environmental cues (Gogvadze et al., 2008; Youle and van der Bliek, 2012; Liesa and Shirihai, 2013; Mishra and Chan, 2014; Chen and Chan, 2017). Mitochondrial fusion is mediated by dynamin family GTPases, such as mitofusin 1 (MFN1) and mitofusin 2 (MFN2), which regulate fusion of mitochondrial outer membranes (Liesa and Shirihai, 2013; Mishra and Chan, 2014; Chen and Chan, 2017). Fusion promotes complementation between damaged mitochondria, while fission enhances elimination of damaged mitochondria by mitophagy in a PARKIN-dependent or -independent manner to maintain mitochondrial fitness (Gogvadze et al., 2008; Youle and van der Bliek, 2012; Chen and Dorn, 2013; Liesa and Shirihai, 2013; Mishra and Chan, 2014; Gong et al., 2015; Lazarou et al., 2015; Chen and Chan, 2017).

Here we show that PKM2 binds to MFN2 and thereby promotes mitochondrial fusion and OXPHOS with concomitant attenuation of glycolysis. The PKM2:MFN2 interaction is enhanced by mTOR-mediated phosphorylation of MFN2. Our results demonstrated that an mTOR-MFN2-PKM2 signaling axis is critical for the metabolic switch between glycolysis and OXPHOS to control cancer cell growth. Therefore, treatments to modulate this switch could be therapeutically effective against cancer.

## Results

### PKM2 promotes mitochondrial OXPHOS

**Figure 1:**
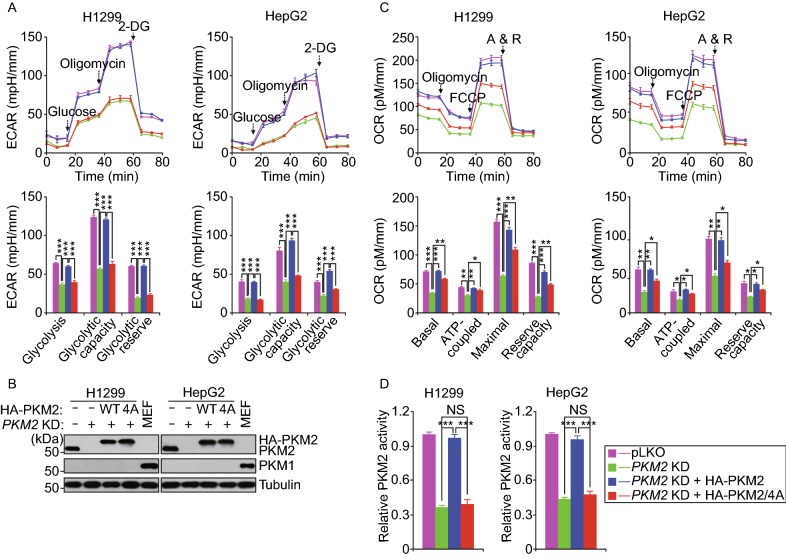
**PKM2 promotes mitochondrial OXPHOS**. (A) Effects of PKM2 on extracellular acidification rate (ECAR) in H1299 and HepG2 cells. Top panels: ECAR traces were obtained using a Seahorse XF96 Analyzer. Bottom panels: Statistical analyses of glycolysis, glycolytic capacity and glycolytic reserve in ECAR. 2-DG, 2-deoxy-D-glucose. (B) Immunoblots showing the expression of wild type (WT) PKM2 or mutants of PKM2 in H1299 and HepG2 cells. The right-hand lane (labeled MEF) contains TCL of mouse embryonic fibroblasts. (C) Effects of PKM2 on oxygen consumption rate (OCR) in H1299 and HepG2 cells. Top panels: OCR traces were obtained using a Seahorse XF96 Analyzer. Bottom panels: Statistical analyses of baseline respiratory capacity, ATP-coupled respiratory capacity, maximum respiratory capacity and reserve respiratory capacity in OCR. FCCP, carbonyl cyanide-4 (trifluoromethoxy) phenylhydrazone. A & R, antimycin plus rotenone. (D) Relative PKM2 activity in H1299 and HepG2 cells. NS, no statistical significance. PKM2/4A is the R73A/K270A/D296A/T328A mutant of PKM2. Data are shown as mean ± s.e.m. *P < 0.05, **P < 0.01, ***P < 0.001, *n* = 6

Although PKM2 is the predominant isoform in most human cancers to catalyze the final step of glycolysis, it is currently unclear whether OXPHOS is affected by PKM2 in human cancer cells. Consistent with its role in glycolysis, PKM2 knockdown decreases glycolytic activity in both H1299 (derived from human lung cancer) and HepG2 (derived from human liver cancer) cells, as judged by measuring the extracellular acidification rate (ECAR) (Fig. 1A and 1B). In addition, knockdown of PKM2 also dramatically decreases OXPHOS as judged by oxygen consumption rate (OCR) (Fig. 1C). Addition of wild type (WT) PKM2 completely restores both glycolysis and OXPHOS activity (Fig. 1A–C).

To determine whether PKM2 has a glycolytic or non-glycolytic effect on mitochondrial respiration, we made a glycolysis-defective mutant of PKM2 (4A, R73A/K270A/D296A/T328A), which cannot bind to PEP (Valentini et al., 2002). PKM2/4A lost its enzyme activity and glycolytic capacity as judged by PKM2 activity and ECAR (Figs. 1A, 1D and S1A–C). We next tested whether this mutant can rescue the effect of PKM2 deficiency on mitochondrial respiration. Compared to WT PKM2, PKM2/4A restores mitochondrial OXPHOS by about 50% (Figs. 1C and S1). These results indicate that PKM2 modulates mitochondrial dynamics in both a glycolysis-dependent and -independent manner.

### PKM2 promotes mitochondrial fusion

**Figure 2:**
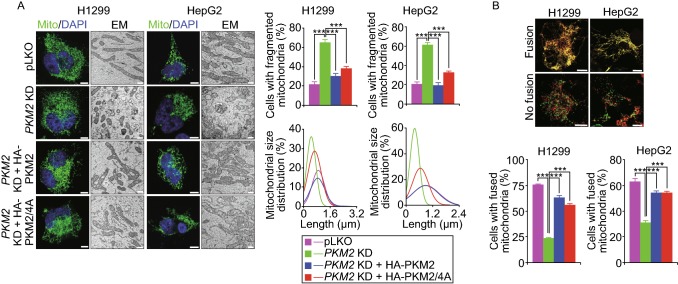
**PKM2 promotes mitochondrial fusion**. (A) Effect of PKM2 on mitochondrial morphology. Left panel: Representative confocal and electron microscopy (EM) images showing the effect of different PKM2 treatments on mitochondrial phenotypes in H1299 and HepG2 cells. In the confocal images, cells are labeled with Green FP-Mitochondrion (Mito). Right panel: Statistical analyses showing the proportion of cells with fragmented mitochondria and the distribution of mitochondrial size. DAPI, 4,6-diamidino-2-phenylindole. *n* = 95–173 cells. (B) Representative images and statistical analyses showing the effect of PKM2 on mitochondrial fusion in H1299 and HepG2 cells. *n* = 150 cells. Scale bars for confocal imaging, 10 μm. Scale bars for EM, 500 nm. PKM2/4A is the R73A/K270A/D296A/T328A mutant of PKM2. Data are shown as mean ± s.e.m. ***P < 0.001

Mitochondrial dynamics is known to affect OXPHOS (Chen et al., 2005; Mourier et al., 2015; Buck et al., 2016). To investigate whether PKM2 regulates OXPHOS by affecting mitochondrial dynamics, mitochondrial morphology was examined in H1299 and HepG2 cells. Knockdown of PKM2 dramatically increases the fragmentation of mitochondria as judged by confocal and electron microscopy (EM) (Fig. 2A). Addition of wild type (WT) PKM2 or the glycolysis-defective mutant PKM2/4A restores mitochondrial morphology (Fig. 2A). These results indicate that PKM2 might promote mitochondrial fusion. To test this notion, a mitochondrial fusion assay was carried out as described (Song et al., 2009). As shown in Fig. 2B, PKM2 knockdown significantly impaired the fusion activity in both H1299 and HepG2 cells.

Most cancer cells undergo the transition from mitochondrial OXPHOS to aerobic glycolysis (Vander Heiden et al., 2009; Hanahan and Weinberg, 2011; Vander Heiden and DeBerardinis, 2017). It has been reported that cancer cells have fragmented mitochondria (Rehman et al., 2012; Zhao et al., 2013), and therefore we wondered whether tumor tissues also have fragmented mitochondria. When we compared the mitochondrial morphology in human normal tissue samples and cancer tissue samples, we found a dramatic increase of mitochondrial fragmentation in lung, liver, colon and breast cancer tissue samples, although the degrees of fragmentation in the cancer samples are different (Fig. S2). Considering that cancer tissues express high levels of PKM2, these results indicate that PKM2 might maintain the balance of mitochondrial fusion and fission to protect mitochondria from over-fragmentation in cancer cells.

### PKM2 binds to MFN2

**Figure 3:**
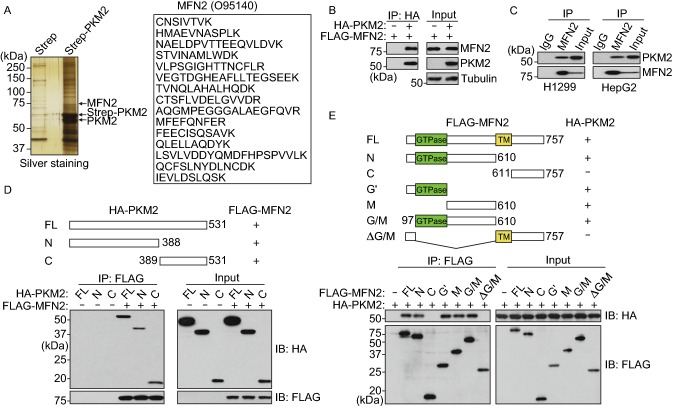
**PKM2 binds to MFN2**. (A) Left panel: Silver-stained gel showing the proteins that interact with Strep-PKM2 in a pull-down assay in HepG2 cells. Right panel: The peptides from MFN2 that were identified by mass spectrometry analysis. (B) Co-immunoprecipitation (co-IP) showing the interaction of overexpressed PKM2 and MFN2 in HEK293T cells. (C) Co-IP showing the interaction of endogenous PKM2 and MFN2 in H1299 and HepG2 cells. (D) Deletion analysis of the regions in PKM2 is required for the PKM2:MFN2 interaction. Interaction-competent PKM2 polypeptides are indicated by (+) in each schematic. (E) Deletion analysis of the regions in MFN2 is required for the PKM2:MFN2 interaction. Interaction-competent MFN2 polypeptides are indicated by (+) in each schematic

To further investigate the non-glycolytic function of PKM2 in mitochondrial fusion, we analyzed the interacting proteins of PKM2 by affinity purification and mass spectrometry identification and found that MFN2 was pulled down by Strep-PKM2 (Fig. 3A). Co-immunoprecipitation assays using both overexpressed and endogenous proteins confirmed that MFN2 binds to PKM2 (Fig. 3B and 3C). In addition, PKM2 has a higher affinity to MFN2 than PKM1, a spliced isoform of pyruvate kinase in non-transformed cells (Fig. S3A). Results from domain mapping showed that the PKM2:MFN2 interaction is mediated through the N-terminus and C-terminus of PKM2 and the N-terminus of MFN2 (Fig. 3D and 3E). This suggests that there are multiple binding sites between PKM2 and MFN2.

### mTOR phosphorylates MFN2 and enhances its binding to PKM2

**Figure 4:**
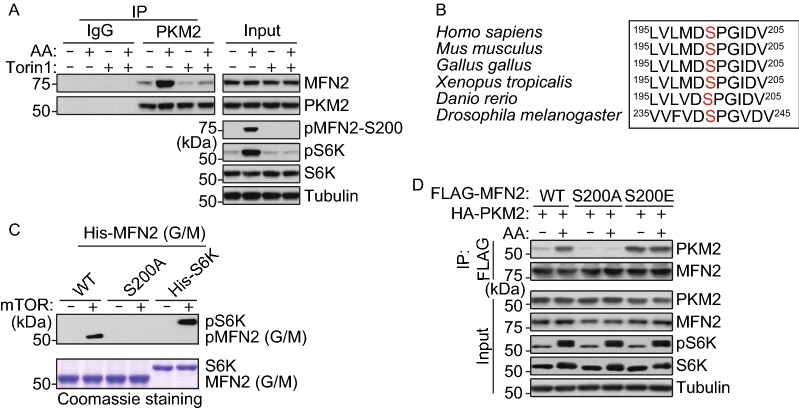
**mTOR phosphorylates MFN2 and enhances its binding to PKM2**. (A) Co-IP showing the interaction of endogenous PKM2 and MFN2 in H1299 cells in response to treatment with amino acids and/or Torin1. H1299 cells incubated with amino acid-free RPMI1640 for 6 h were exposed to 250 nmol/L Torin1 or control vehicle for another 2 h, and then treated with amino acids for 30 min. (B) Amino acid sequence alignment of MFN2 orthologs. The conserved serine residue (S, red) is phosphorylated by mTOR. (C) *In vitro* kinase assay showing phosphorylation of the His-tagged G/M fragment of MFN2 (which contains amino acids 97–610 of MFN2) and S6K by truncated mTOR. (D) Immunoblots of co-IP assays showing the relative association of PKM2 with WT MFN2 or MFN2 with a Ser-to-Ala or Ser-to-Glu mutation at position 200 (S200A or S200E) in transfected HEK293T cells in the presence or absence of amino acids

Interestingly, the association of PKM2 and MFN2 is enhanced by treatment with amino acids or FBS (fetal bovine serum) and blocked by pre-incubation with the mTOR inhibitors Torin1 or rapamycin (Figs. 4A, S3B and S3C), which indicates that mTOR promotes the PKM2:MFN2 interaction. In addition, the glycolysis-defective mutant of PKM2 has a similar response to amino acid stimulation, although it has a stronger binding to MFN2 than WT PKM2 (Fig. S3D). The PKM2:MFN2 association was blocked by knockout of *Raptor* (a subunit of mTORC1 (Saxton and Sabatini, 2017)) but not by knockout of *Rictor* (a subunit of mTORC2 (Saxton and Sabatini, 2017)) (Fig. S3E), which indicates that mTORC1 is in charge of the phosphorylation of MFN2. mTOR interacts with MFN2 and mTOR inhibition dramatically increases mitochondrial fragmentation (Fig. S3F and S3G).

To test how mTOR mediates the PKM2:MFN2 association, we checked the potential mTOR phosphorylation sites in PKM2 and MFN2 by amino acid scanning (Fig. S3H and S3I). There is a conserved serine site at position 200 (S200) of MFN2 (Fig. 4B), which occurs in the context of a classic mTOR substrate motif (S/T-P) (Hsu et al., 2011). An *in vitro* kinase assay showed that mTOR directly phosphorylates MFN2 at Ser200 (Fig. 4C). To confirm the phosphorylation of MFN2 at Ser200 *in vivo*, we raised a polyclonal antibody against the phospho-MFN2 (Ser 200) peptide and found that MFN2 was phosphorylated in response to amino acid treatment and dephosphorylated in the presence of Torin1 (Figs. 4A and S3J). These results suggest that MFN2 is a *bona fide* substrate of mTOR.

The S200A mutant of MFN2, which cannot be phosphorylated by mTOR, had a similar cellular localization to WT MFN2, but the amino acid-stimulated enhancement of PKM2:MFN2 association was blunted by this mutant (Figs. 4D and S3K). In contrast, the phospho-mimic mutant (S200E) of MFN2 constitutively increased the association (Figs. 4D and S3K).

### Phosphorylation of MFN2 enhances mitochondrial OXPHOS and attenuates glycolysis

**Figure 5:**
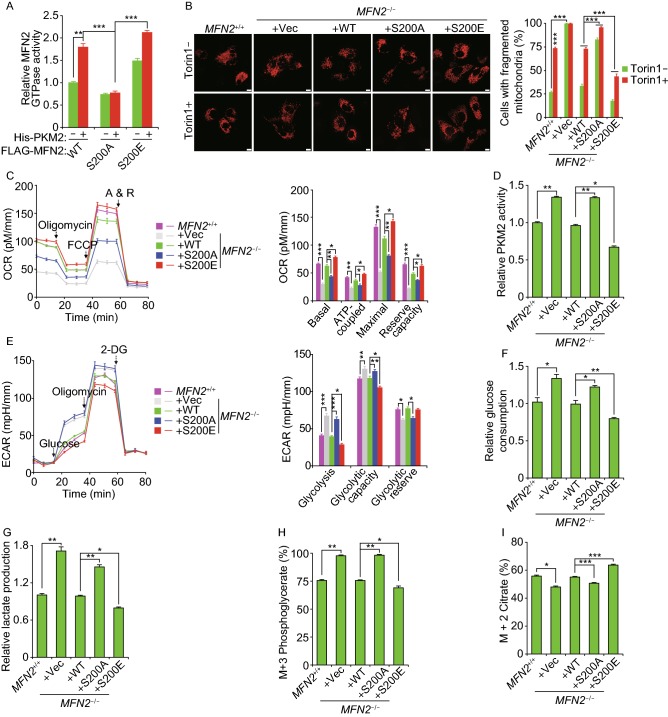
**Phosphorylation of MFN2 enhances mitochondrial OXPHOS and attenuates glycolysis**. (A) Effect of PKM2 on the GTPase activity of WT MFN2 or mutants of MFN2. (B) Representative images and statistical analyses showing the effect of MFN2 and its mutants on mitochondrial morphology in H1299 cells in the presence or absence of Torin1 (250 nmol/L) for 4 h. (C–I) Effect of MFN2 and its mutants on OCR (C), relative PKM2 activity (D), ECAR (E), relative glucose consumption (F), relative lactate production (G), ratio of M+3 2-phosphoglycerate/3-phosphoglycerate (H) and M+2 citrate (I). Data are shown as mean ± s.e.m. *P < 0.05, **P < 0.01, ***P < 0.001, *n* = 6

We first investigated the role of PKM2:MFN2 association on MFN2 GTPase activity. Wild type (WT) and mutated FLAG-MFN2 were immunoprecipitated from *Mfn1*^−/−^ and *Mfn2*^−/−^ double knockout MEF cells with stable expression of human-sourced WT and mutated MFN2 and were incubated in the presence or absence of purified His-PKM2 from *E*. *coli*. (Fig. S4A and S4B). The GTPase activity of WT MFN2 but not the MFN2/S200A mutant was increased by PKM2, while the MFN2/S200E mutant had slightly stronger GTPase activity than WT MFN2 (Fig. 5A).

Next, we investigated the roles of PKM2:MFN2 association and its modulation by mTOR in mitochondrial morphology and OXPHOS. The MFN2/S200A mutant increased mitochondrial fragmentation, while MFN2/S200E was resistant to Torin1-induced mitochondrial fragmentation (Fig. 5B). As measured by OCR, MFN2/S200E restored OXPHOS more efficiently than WT MFN2, while the MFN2/S200A mutant only partially restored OXPHOS in *Mfn2*^−/−^ cells (Fig. 5C).

Since mTOR modulates mitochondrial morphology and OXPHOS by phosphorylating MFN2, we further investigated whether phosphorylated MFN2 also affects glycolysis through binding with PKM2. PKM2 activity, ECAR and metabolic tracing of glycolytic flux were measured in *Mfn2*^−/−^ knockout cells expressing WT or mutant forms of MFN2. PKM2 activity and PKM2-dependent glycolytic function were enhanced by MFN2 knockout and repressed by adding WT MFN2 or the MFN2/S200E mutant but not MFN2/S200A (Figs. 5D–I and S4C). Furthermore, compared to WT MFN2, the S200E mutant of MFN2 had an even better rescuing effect on OCR and glycolytic function (Fig. 5C–I). Together, these results demonstrate that phosphorylation of MFN2 mediated by mTOR enhances mitochondrial fusion and OXPHOS and attenuates glycolysis.

### The phosphorylation status of MFN2 affects cancer cell growth

**Figure 6:**
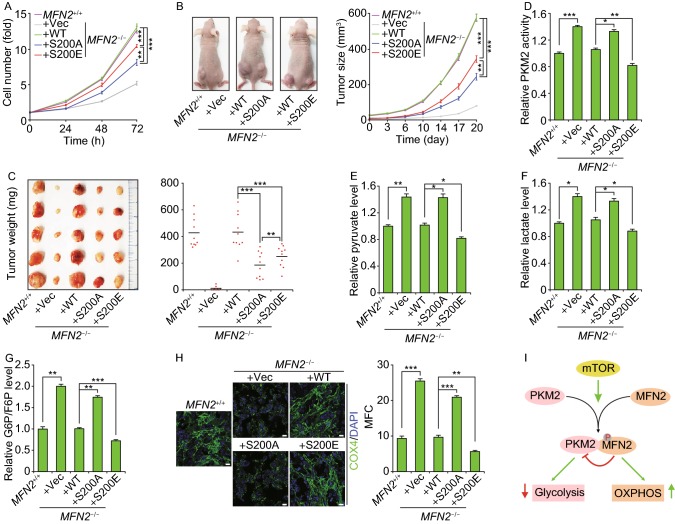
**The phosphorylation status of MFN2 affects cancer cell growth**. (A) Effect of MFN2 and its mutants on cell proliferation in H1299 cells. *n* = 6. (B and C) Representative images and statistical analyses showing the effect of MFN2 and its mutants on tumor size (B) or tumor weight (C) in nude mice. *n* = 10 mice. (D–G) Effect of MFN2 and its mutants on relative PKM2 activity (D), relative pyruvate level (E), relative lactate production (F) and relative G6P/F6P (glucose-6-phosphate/fructose-6-phosphate) level (G) in tumors from the xenograft assay. (H) Representative images and statistical analyses showing the effect of MFN2 and its mutants on mitochondrial morphology in tumors from the xenograft assay. Scale bars, 10 μm. (I) mTOR phosphorylates MFN2 at Ser200, which enhances the interaction of MFN2 with PKM2, promotes OXPHOS by increasing mitochondrial fusion, and attenuates glycolysis. Data are shown as mean ± s.e.m. *P < 0.05, **P < 0.01, ***P < 0.001

Since the phosphorylation-defective MFN2 mutants affect both OXPHOS and glycolysis, we evaluated the effect of WT MFN2 and mutants of MFN2 on the growth of H1299 cancer cells using cell proliferation assays and xenograft assays in nude mice. The growth of untreated *MFN2*^−/−^ cells was greatly reduced compared to *MFN2*^+/+^ cells, but was restored by addition of WT MFN2 (Figs. 6A–C, S4D and S4E). Both the S200A and S200E mutants of MFN2 partially restored cancer cell growth (Figs. 6A–C, S4D and S4E). Mechanistically, PKM2 activity, PKM2-dependent glycolytic function and mitochondrial fragmentation in tumors were enhanced by MFN2 knockout and repressed by adding WT MFN2 or the MFN2/S200E mutant but not the MFN2/S200A mutant (Fig. 6D–H). These results are similar to those in H1299 cells (Fig. 5). Furthermore, compared to WT MFN2, the S200E mutant of MFN2 had an even stronger rescuing effect on glycolytic function and mitochondrial dynamics (Fig. 6D–H). These results suggest that neither the enhancement of mitochondrial fission by *MFN2*^−/−^ or addition of S200A, nor the enhancement of mitochondrial fusion and OXPHOS by S200E, is fully favorable for cancer cell growth. Together, these results show that phosphorylation of MFN2 affects cancer cell growth by promoting mitochondrial fusion and OXPHOS and suppressing glycolysis. Thus, the mTOR-MFN2-PKM2 signaling axis coordinates glycolysis and OXPHOS to modulate cancer cell growth.

## Discussion

Previous results have shown that pyruvate dehydrogenase and lactate dehydrogenase regulate the switch between glycolysis and OXPHOS by controlling the flux from glycolysis to the tricarboxylic acid cycle and inducing passive mitochondrial adaptation (Fantin et al., 2006; Gogvadze et al., 2008). In this study, we demonstrate that mTOR phosphorylates MFN2 and thereby enhances its interaction with PKM2 and further modulates the switch between glycolysis and OXPHOS (Fig. 6I). These results clarify the effect of an mTOR-MFN2-PKM2 signaling axis on this switch and expand the list of regulatory factors that are involved in this process. In addition, our results broaden the non-glycolytic roles of PKM2 in cancer cell growth (Israelsen et al., 2013; Yang and Lu, 2015; Dayton et al., 2016). Considering the role of AMPK as a sensor of energy stress and mitochondrial damage, the seamless response of PKM2, mTOR and AMPK to different environmental cues will guarantee the dynamic flow of glycolysis, OXPHOS and mitochondrial fusion/fission to maintain mitochondrial fitness for proper cell growth (Youle and van der Bliek, 2012; Cornu et al., 2013; Yang and Lu, 2015; Dayton et al., 2016; Garcia and Shaw, 2017; Saxton and Sabatini, 2017; Zhang et al., 2017).

Compared to PKM1, which is tetrameric and has higher constitutive activity, PKM2 acts as a monomer or dimer, has lower glycolytic activity, and is modulated by allosteric activators (Christofk et al., 2008a; Christofk et al., 2008b; Anastasiou et al., 2012; Chaneton et al., 2012; Keller et al., 2012). In the future, it will be interesting to test whether the allosteric activators of PKM2 affect its association with MFN2. Our results show that MFN2 can interact with both PKM1 and PKM2 with different binding affinity (Fig. S3A). Considering that PKM1 but not PKM2 exists in normal cells (Christofk et al., 2008a; Yang and Lu, 2015; Dayton et al., 2016), it remains to be confirmed in normal cells whether the interaction of PKM1 and MFN2 has a similar regulatory effect on glycolysis and OXPHOS.

It is well established that both glycolysis and mitochondrial dynamics are important for cancer cell growth (Gogvadze et al., 2008; Vander Heiden et al., 2009; Hanahan and Weinberg, 2011; Youle and van der Bliek, 2012; Liesa and Shirihai, 2013; Mishra and Chan, 2014; Serasinghe et al., 2015; Chen and Chan, 2017). Mitochondrial fragmentation is increased in cancer cells, while the increased levels of pS200 in cancer tissues suggest enhanced mitochondrial fusion (Figs. S2 and S5). In addition, our results showed that PKM2 knockdown in cancer cells further enhanced mitochondrial fragmentation (Fig. 2). These results suggest that mitochondria are in an elongated state in normal cells, in an intermediate state in cancer cells, and in a much more fragmented state in PKM2 knockdown cancer cells. It is possible that PKM2’s pro-fusion activity and the increased levels of pS200 in cancer tissues provide a self-protective mechanism for maintaining the balance of mitochondrial fusion and fission to avoid over-fragmented mitochondria, while also modulating the switch between OXPHOS and glycolysis. These ideas are supported by the results showing that both phospho-defective and phospho-mimic mutants of MFN2 suppress cancer cell growth (Fig. 6). In summary, our study reveals a novel signaling axis composed of mTOR, MFN2 and PKM2, which controls the metabolic switch between glycolysis and OXPHOS for cancer cell growth. The mechanism revealed by our results may provide a new therapeutic approach against cancer.

## Materials and Methods

### Plasmids

Myc-DDK-PKM1 (RC219382) and Myc-DDK-PKM2 (RC201855) were purchased from OriGene. Green FP-Mitochondrion (558718) and Red FP-Mitochondrion (558722) were from BD Pharmingen^TM^. Myc-mTOR (1861) was purchased from Addgene. MFN2 was amplified from a HEK293T cDNA library. The specific shRNA sequence targeting only PKM2 was as follows: 5′-CTACCACTTGCAATTATTTGA-3′. The PKM2 knockdown-resistant plasmid was obtained by nonsense mutation. Site-directed mutagenesis was performed with the QuikChange strategy (Agilent Technologies). All expressed constructs used in this study were confirmed by sequencing.

### Cell culture, transfection and viral infection

MEF, HEK293T (ATCC), HepG2 (ATCC) and H1299 (ATCC) cells were cultured in DMEM, DMEM, MEM and RPMI1640 respectively, containing 10% FBS (HyClone) and 100 mg/mL penicillin-streptomycin. Mouse primary hepatocytes were isolated as previously described (Han et al., 2015) and cultured in M199 medium. *Raptor*^*fl*/*fl*^ (013188) *and Rictor*^*fl*/*fl*^ (020649) mice were from the Jackson Laboratory and were bred to homozygosity. For generation of liver-specific *Raptor* or *Rictor* knockout mice, *Raptor*^*fl*/*fl*^ or *Rictor*^*fl*/*fl*^ mice were crossed with *Alb*-Cre mice. Wild type and *Mfn1*^−/−^/*Mfn2*^−/−^ MEF cells were kindly provided by Dr. Quan Chen (Institute of Zoology, Chinese Academy of Sciences) and originally by Dr. David Chan (California Institute of Technology). Cells were transfected with Lipofectamine 2000^®^ (Thermo Fisher Scientific) following the manufacturer’s protocol. Lentiviruses were generated by co-transfecting HEK293T cells with pLKO.1-puro (empty vector or PKM2 shRNA), VSVG, PMDL and REV. Retroviruses were generated by co-transfecting HEK293T cells with plasmids expressing PKM2 or MFN2, VSVG and Hit-60. H1299, HepG2 and MEF cells were infected by lentiviruses and/or retroviruses with 8 μg/mL polybrene and selected in 2 μg/mL puromycin and/or 350 μg/mL hygromycin for at least 7 days. All cell lines were routinely tested for mycoplasma using a PCR detection kit (MP0035, Sigma-Aldrich).

### Generation of *MFN2* knockout cell lines using CRISPR/Cas9 gene editing

The short guide RNAs targeting exon 3 of the human *MFN2* gene (NG_007945.1) were designed (http://crispr.mit.edu/) and synthesized to make the PX459-sgRNA-Cas9 constructs. In brief, the 25-bp DNA oligos containing the 20-bp target sequence and *Bbs1* sticky ends were annealed and inserted into the PX459 plasmid (48139, Addgene) digested with *Bbs1*. The DNA sequences for generating sgRNA were as follows: forward: 5′-CACCGGCAGCTGGGGGCCTACATCC-3′ and reverse: 5′-AAACGGATGTAGGCCCCCAGCTGCC-3′. For the control plasmid, no sgRNA sequence was inserted into the construct. *MFN2*-edited cells and control cells were selected by using 4 μg/mL puromycin. To determine the genome-editing effect, genomic DNA was extracted from the *MFN2*-edited cells and control cells, and analyzed by sequencing the PCR amplification products of the edited region. The PCR primers were as follows: forward: 5′-AGAGACACATGGCTGAGGTG-3′ and reverse: 5′-GCCTGGCATGTAAAACAACG-3′. The editing of *MFN2* was further validated by immunoblots.

### Cell proliferation and viability assays

5 × 10^4^ H1299 or HepG2 cells were seeded in triplicate in 6-well plates, and accurate cell counts were obtained every 24 h for a 4-day period. Time zero was taken as 16 h after seeding. Cell viability was measured according to the manufacturer’s instructions (ENZ-51002, Enzo).

### Oxidative phosphorylation and glycolysis assays

The intact cellular oxygen consumption rate (OCR) and extracellular acidification rate (ECAR) were measured in real time using the Seahorse XF96 Extracellular Flux Analyzer (Seahorse Bioscience). Briefly, 1.0 × 10^4^ H1299 or 2.0 × 10^4^ HepG2 cells were seeded into 96-well Seahorse microplates in 80 μL of growth medium and incubated at 37 °C in 5% CO_2_ for 16 h and the calibrator plate was equilibrated overnight in a non-CO_2_ incubator. Before starting the test, cells were washed twice with assay running media (unbuffered DMEM, 25 mmol/L glucose, 1 mmol/L glutamine, 1 mmol/L sodium pyruvate for OCR; unbuffered DMEM, 1 mmol/L glutamine for ECAR) and equilibrated in a non-CO_2_ incubator. Once the probe calibration was completed, the probe plate was replaced by the cell plate. The protocol was optimized for the simultaneous measurement of OCR and ECAR. For OCR, the analyzer plotted the value as the cells were treated by sequential injection of the following compounds: oligomycin (1 μmol/L), carbonyl cyanide-4 (trifluoromethoxy) phenylhydrazone (FCCP, 0.5 μmol/L), and antimycin A (1 μmol/L) plus rotenone (1 μmol/L). For ECAR, the analyzer plotted the value as the cells were treated by sequential injection of the following compounds: glucose (10 mmol/L), oligomycin (1 μmol/L) and 2-deoxy-glucose (2-DG, 100 mmol/L).

### Mitochondrial fusion assay

The assay was performed as described previously (Song et al., 2009). In brief, cells expressing Green FP-Mitochondrion were seeded into 6-well plates and cultured on coverslips overnight with the same number of cells expressing Red FP-Mitochondrion. The cells were fused for 60 seconds with 50% PEG1500 (Roche) the next morning, then washed thoroughly with 1 × PBS, and cultured for 7 h in medium containing 20 μg/mL cycloheximide before fixation.

### Measurement of pyruvate kinase activity

A total of 1 × 10^5^ cells per well were seeded in 6-well plates for 6 h and then incubated in fresh media for one more day. Cellular pyruvate kinase activities were determined with a Pyruvate Kinase Activity Assay kit (MAK072, Sigma-Aldrich). All the values were normalized to cell numbers. WT PKM2 and PKM/4A were expressed and purified from *E*. *coli* and the pyruvate kinase activity was measured using kinetic analysis as previously described (Wang et al., 2001). The assay includes 10 nmol/L WT PKM2 or PKM2/4A in 25 μL of assay buffer (50 mmol/L Tris, pH7.5, 100 mmol/L KCl, 10 mmol/L MgCl_2_, 3% DMSO) and various concentrations of PEP and ADP. Kinetic parameters were determined for PEP at a fixed concentration of 1.5 mmol/L ADP, and for ADP at 5 mmol/L PEP. In all cases, the reaction was started by adding PEP, and the enzyme activity was assayed at 8 different concentrations of substrate (PEP or ADP).

### Measurement of MFN2 GTPase activity

Wild type (WT) and mutated FLAG-MFN2 were immunoprecipitated from *Mfn1*^−/−^ and *Mfn2*^−/−^ double knockout MEF cells with stable expression of human-sourced WT and mutated MFN2 and were incubated in the presence or absence of purified His-PKM2 from *E*. *coli*. The GTPase activity of MFN2 immunoprecipitates was examined with a GTPase Activity Assay kit (MAK113, Sigma-Aldrich).

### *In vitro* kinase assay

His-FLAG-tagged G/M wildtype and S200A mutant of MFN2 (amino acids 97–610), and S6K fusion proteins were purified from *E*. *coli*. The kinase assay was performed as reported (Han et al., 2015). The reaction system (20 μL), containing 150 ng fusion protein, 20 ng truncated mTOR (Millipore, 14-770) in reaction buffer (25 mmol/L HEPES pH 7.4, 50 mmol/L KCl, 5 mmol/L MgCl_2_, and 5 mmol/L MnCl_2_), and 50 μmol/L cold ATP, was incubated for 30 min at 30 °C. Reactions were stopped by adding 5 μL sample buffer, then boiled for 10 min and analyzed by SDS-PAGE followed by detection with antibodies against pMFN2 (S200) and pS6K (9234, Cell Signaling Technology).

### Immunostaining and image analyses

Human frozen tissue arrays (FMC402d, FMC282d, FLV401a, BRF404b, FLU401a, FCO405a, AlenaBio) were fixed with precooled methanol for 5 min at − 20 °C. For immunostaining of H1299 and HepG2, cells were fixed in 4% PFA for 10 min. Fixed cells or cryostat sections were permeabilized in 0.2% Triton X-100 and blocked in 5% BSA for 1 h, and then incubated with DAPI, or the appropriate primary and secondary antibodies. Samples were mounted with mounting medium (C9368, Sigma). Images were acquired with a deconvolution microscope (Zeiss). Mitochondrial fragmentation count (MFC) was acquired as reported (Rehman et al., 2012).

### Electron microscopy

Cells were fixed in 2.5% glutaraldehyde in 0.1 mol/L MOPS buffer (pH 7.0) for 8 h at room temperature, then 2.5% glutaraldehyde and 1% paraformaldehyde in 0.1 mol/L MOPS buffer (pH 7.0) for 16 h at 4 °C. They were then post-fixed in 1% osmium tetroxide for 1 h, embedded in Spurr’s resin, sectioned, doubly stained with uranyl acetate and lead citrate, and analyzed using a Hitachi H7650B transmission electron microscope. Mitochondrial length (150 mitochondria) was measured by placing a tip-to-tip line across the longest axis of each mitochondrion using the straight-line tool in ImageJ.

### Immunoblotting and immunoprecipitation

Assays were performed as described previously (Han et al., 2015; Chen et al., 2017). In brief, cells were lysed in cold cell lysis buffer (HEPES pH 7.4 50 mmol/L, NaCl 150 mmol/L, Triton X-100 1% and glycerol 10%) supplemented with phosphatase and protease inhibitors (B14011 and B15001, Bimake), sonicated and centrifuged for 15 min at 15,000 rpm at 4 °C. Total protein (20 μg) from each lysate was separated by SDS-PAGE, transferred onto a nitrocellulose membrane, and probed with the indicated antibodies. For immunoprecipitation, control IgG or antibody was incubated overnight at 4 °C with supernatant. Antibodies were purchased as follows: mouse monoclonal anti-HA antibody (MMS-101P), Covance; mouse monoclonal anti-FLAG antibody (F1804) and anti-Tubulin (T5201), Sigma-Aldrich; rabbit polyclonal anti-HA (561) and anti-FLAG (PM020), MBL International; anti-MFN2 (ab56889) and anti-COX4 (ab14744), Abcam; anti-PKM2 (4053), anti-S6K (2708s) and anti-pS6K (9234), Cell Signaling Technology; anti-Myc (SC-40), Santa Cruz. The phospho-S200 MFN2 antibody was generated and purified by AbMax Biotechnology.

### Xenograft studies

Nude mice (nu/nu; 6 weeks old) were used for *in vivo* studies. A suspension of 3 × 10^6^ H1299 cells (in 0.1 mL PBS) was inoculated subcutaneously into the flanks of each mouse. Tumors were measured twice weekly using calipers, and volume was calculated as length × width^2^ × 0.5. At 6 weeks after injection, the tumors were dissected and weighed. Mice were housed in colony cages with a 12-h light/dark cycle in a temperature-controlled environment. All mouse experiments were approved by the Animal Care and Use Committee at Tsinghua University.

### Glycolytic flux measurements

The glycolytic flux was measured based on the rate of glucose consumption and the ratio of ^13^C incorporated into lactate determined by LC-MS. Briefly, cells were cultured in medium with or without [U-^13^C6] glucose. After 12 h, medium was collected and cells were treated with cold 80% methanol. Metabolites were extracted and analyzed by LC-MS. Flux analysis was performed on a TSQ Quantiva Triple Quadrupole mass spectrometer (Thermo Fisher) with positive/negative ion switching. MRM mode was used for data acquisition. Mobile phase A was prepared by adding 2.376 mL tributylamine and 0.858 mL acetic acid to HPLC-grade water, then added HPLC-grade water to give a total volume of 1 liter. Mobile phase B was HPLC-grade methanol. Polar metabolites were separated on a Synergi Hydro-RP 100A column with the column temperature at 35 °C. The measured mass isotopomer distributions were corrected according to their natural abundances.

### Mass spectrometry (MS)

To identify PKM2-interacting proteins, HepG2 cells stably expressing Strep-PKM2 were generated. Immunoprecipitates of Strep-PKM2 were analyzed by electrospray ionization tandem MS on a Thermo LTQ Orbitrap instrument as previously described (Han et al., 2015; Chen et al., 2017).

### Statistical analyses

Age- and weight-matched male mice were randomly assigned for the experiments. The animal numbers used for all experiments are outlined in the corresponding figure legends. No animals were excluded from statistical analyses, and the investigators were not blinded in the studies. No statistical methods were used to predetermine sample size. All studies were performed on at least three independent occasions. Results are reported as mean ± s.e.m. Comparison of different groups was carried out using two-tailed unpaired Student’s *t*-test or one-way ANOVA. Differences were considered statistically significant at *P* < 0.05.

## Electronic supplementary material

Below is the link to the electronic supplementary material.

Electronic supplementary material 1 (PDF 29044 kb)
