# Supplementary material for: PKM2 coordinates glycolysis with mitochondrial fusion and oxidative phosphorylation
Source: Protein Cell. 2019 Mar 18;10(8):583–94. doi: 10.1007/s13238-019-0618-z (PMC6626593; doi:10.1007/s13238-019-0618-z)

SUPPLEMENTARY INFORMATION

Figure S1: Effect of PKM2 on cell proliferation and cell viability.

(A) Coomassie-stained gel showing the recombinant His-PKM2 and His-PKM2/4A used for *in vitro* measurement of PKM2 activity. His-PKM2 and His-PKM2/4A were purified from *E. coli*. PKM2/4A is the R73A/K270A/D296A/T328A mutant of PKM2.

(B) Kinetics of recombinant PKM2 and PKM2/4A as a function of PEP. The ADP concentration was fixed at 1.5 mM.

(C) Kinetics of recombinant PKM2 and PKM2/4A as a function of ADP. The PEP concentration was fixed at 5 mM.

(D-E) Effect of PKM2 on cell proliferation **(D)** and cell viability **(E)** in H1299 and HepG2 cells.

PKM2/4A is the R73A/K270A/D296A/T328A mutant of PKM2. Data are shown as mean ± s.e.m. ***$P < 0.001$, n = 6.

Figure S2: Enhanced mitochondrial fragmentation in human tumor samples.

Representative confocal images showing mitochondrial morphology in human normal (NT) and cancer (CT) tissues (left panel)**.** Samples are labeled with an antibody against COX4 (cytochrome c oxidase subunit 4), which is located in the inner mitochondrial membrane. Boxed areas are shown enlarged in the adjacent panels. Right panel: Statistical analyses of the mitochondrial fragmentation count (MFC).

Scale bars, 10 μm. Data are shown as mean ± s.e.m. ***$P < 0.001$, n = 5 patients for lung, liver and breast NT; n = 20 patients for colon NT; n = 20 patients for liver, colon and breast CT; n = 40 patients for lung CT.

Figure S3: Association of PKM2 with MFN2.

(A) Immunoblots of co-IP assays showing the interaction of MFN2 with PKM1 and PKM2 in *PKM2*-depleted HEK293T cells.

(B) Immunoblots of co-IP assays showing the interaction of PKM2 and MFN2 in HEK293T cells in the presence or absence of 10% FBS.

(C) Immunoblots of co-IP assays showing the effect of rapamycin on the PKM2:MFN2 association in HEK293T cells.

(D) Immunoblots of co-IP assays showing the interaction of WT PKM2 and the glycolysis-defective mutant of PKM2 (4A) with MFN2 in HEK293T cells in the presence or absence of amino acids.

(E) Immunoblots of co-IP assays showing the interaction of PKM2 and MFN2 in mouse primary hepatocytes in the presence or absence of 10% FBS.

(F) Immunoblots of co-IP assays showing the interaction of mTOR and MFN2 in HEK293T cells.

(G) Representative images and statistical analyses showing the effect of mTOR on mitochondrial morphology in H1299 and HepG2 cells. H1299 and HepG2 cells transfected by Red FP-mitochondrion (Mito) were cultured in the

presence or absence of Torin1 (250 nmol/L) or Rapamycin (100 nmol/L) for 4 h.
(H) Immunoblots of co-IP assays showing the interaction of MFN2 with WT PKM2 and mutants of PKM2 in HEK293T cells.
(I) Immunoblots of co-IP assays showing the interaction of PKM2 with WT MFN2 and mutants of MFN2 in HEK293T cells.
(J) Immunoblots showing the phosphorylation status of WT MFN2 and MFN2/S200A in HEK293T cells in response to amino acid treatment for 30 min.
(K) Representative images showing the cellular localization of WT MFN2, MFN2/S200A and MFN2/S200E in HEK293T cells.
Scale bars, 10 μm. Data are shown as mean ± s.e.m. ****P* < 0.001, n = 150 cells.

Figure S4: Evaluation of MEF and H1299 cells stably expressing MFN2 and its mutants.
(A) Immunoblots showing the expression of MFN2 and its mutants in *Mfn1*$^{-/-}$/*Mfn2*$^{-/-}$ double knockout MEF cells.
(B) Silver-stained gel showing the amounts of MFN2 and His-PKM2 for *in vitro* measurement of MFN2 GTPase activity. FLAG-tagged MFN2 and its mutants were purified from *Mfn1*$^{-/-}$/*Mfn2*$^{-/-}$ double knockout MEF cells, while His-PKM2 was purified from *E. coli*.
(C) Immunoblots showing the expression of MFN2 and its mutants in *MFN2*$^{-/-}$ H1299 stable cell lines.
(D) Effect of MFN2 and its mutants on the viability of H1299 cells.
(E) Immunoblots of xenograft tumor lysates showing the expression of MFN2 and its mutants in nude mice. Xenograft tumors were derived from H1299 stable cell lines.

Figure S5: Enhanced phosphorylation of MFN2 Ser200 in human tumor samples.
Representative images (left panel) and statistical analyses (right panel) showing the relative levels of phosphorylated MFN2 (pS200) and total MFN2 in human normal (NT) and cancer (CT) tissues.
Data are shown as mean ± s.e.m. ****P* < 0.001. n = 5 patients for lung, liver and breast NT; n = 20 patients for colon NT; n = 20 patients for liver, colon and breast CT; n = 40 patients for lung CT.

Figure S1

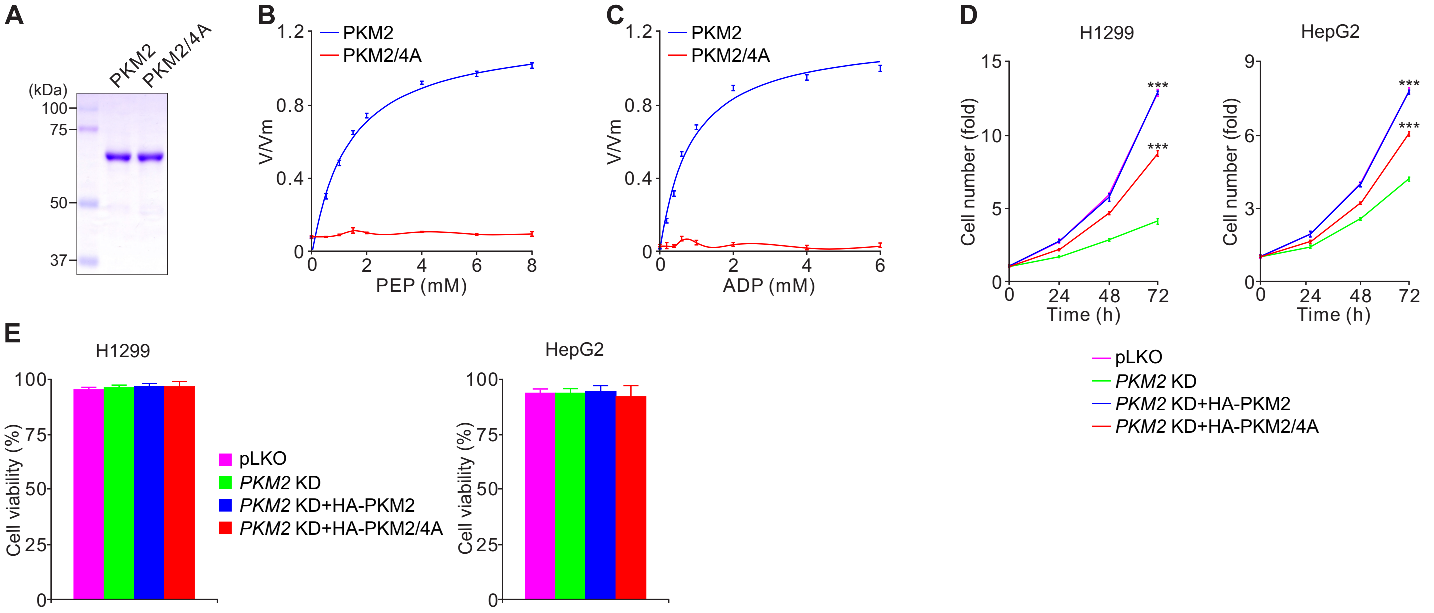

Figure S2

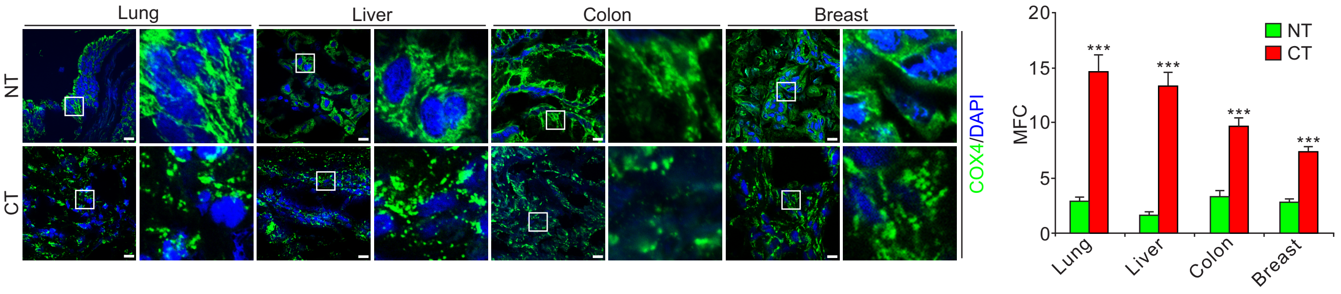

Figure S3

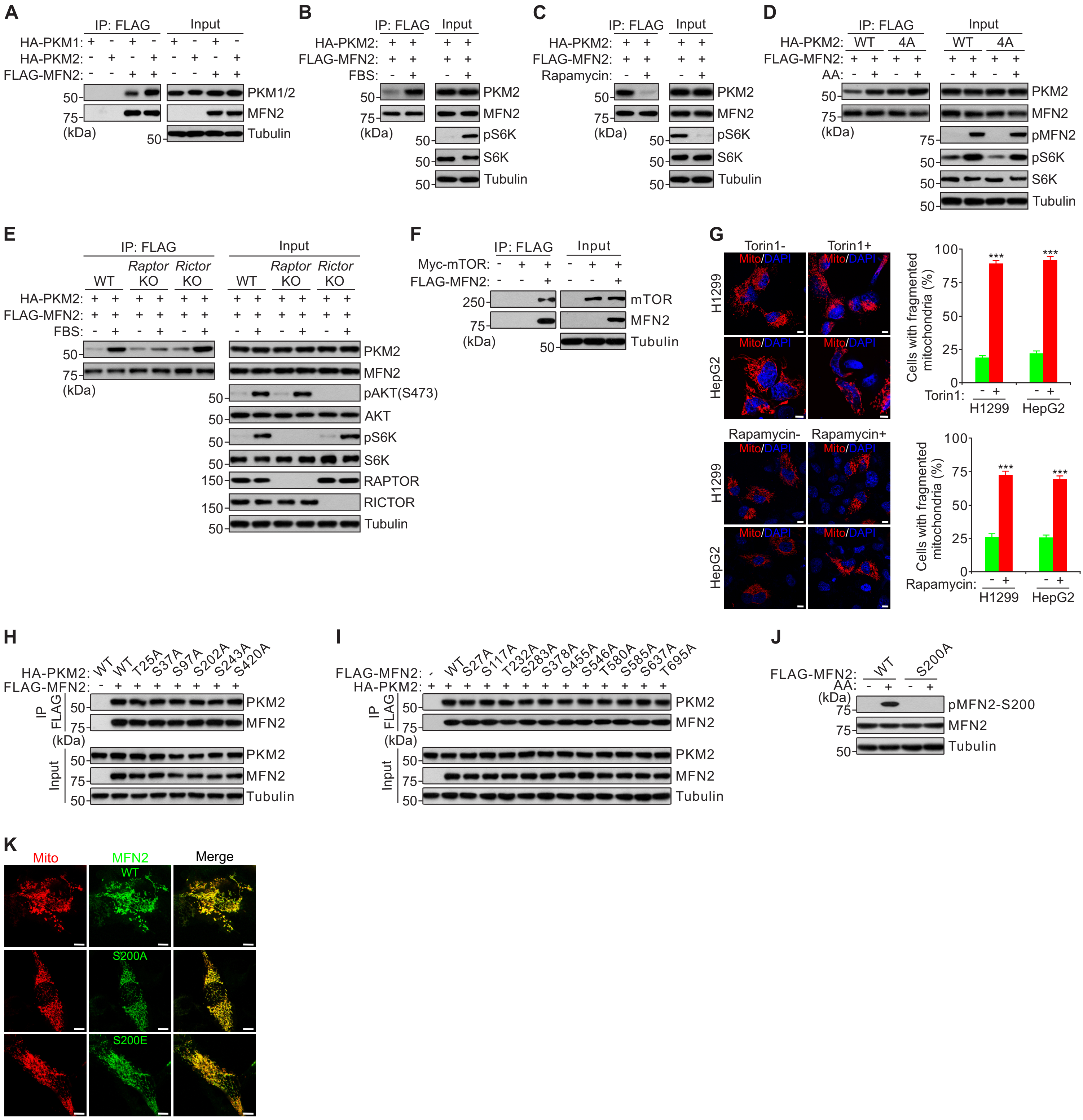
A
IP: FLAG
Input
HA-PKM1:
HA-PKM2:
FLAG-MFN2:
PKM1/2
MFN2
Tubulin
(kDa)
B
FBS:
pS6K
S6K
C
Rapamycin:
D
WT
4A
AA:
pMFN2
E
Raptor KO
Rictor KO
pAKT(S473)
AKT
RAPTOR
RICTOR
F
Myc-mTOR:
mTOR
G
Torin1-
Torin1+
Rapamycin-
Rapamycin+
Mito/DAPI
H1299
HepG2
Cells with fragmented mitochondria (%)
Torin1:
H
HA-PKM2: WT WT T25A S37A S97A S202A S243A S420A
I
FLAG-MFN2: - WT S27A S117A T232A S283A S378A S455A S546A T580A S585A S637A T695A
J
FLAG-MFN2: WT S200A
pMFN2-S200
K
Mito
MFN2
Merge
S200A
S200E

Figure S4

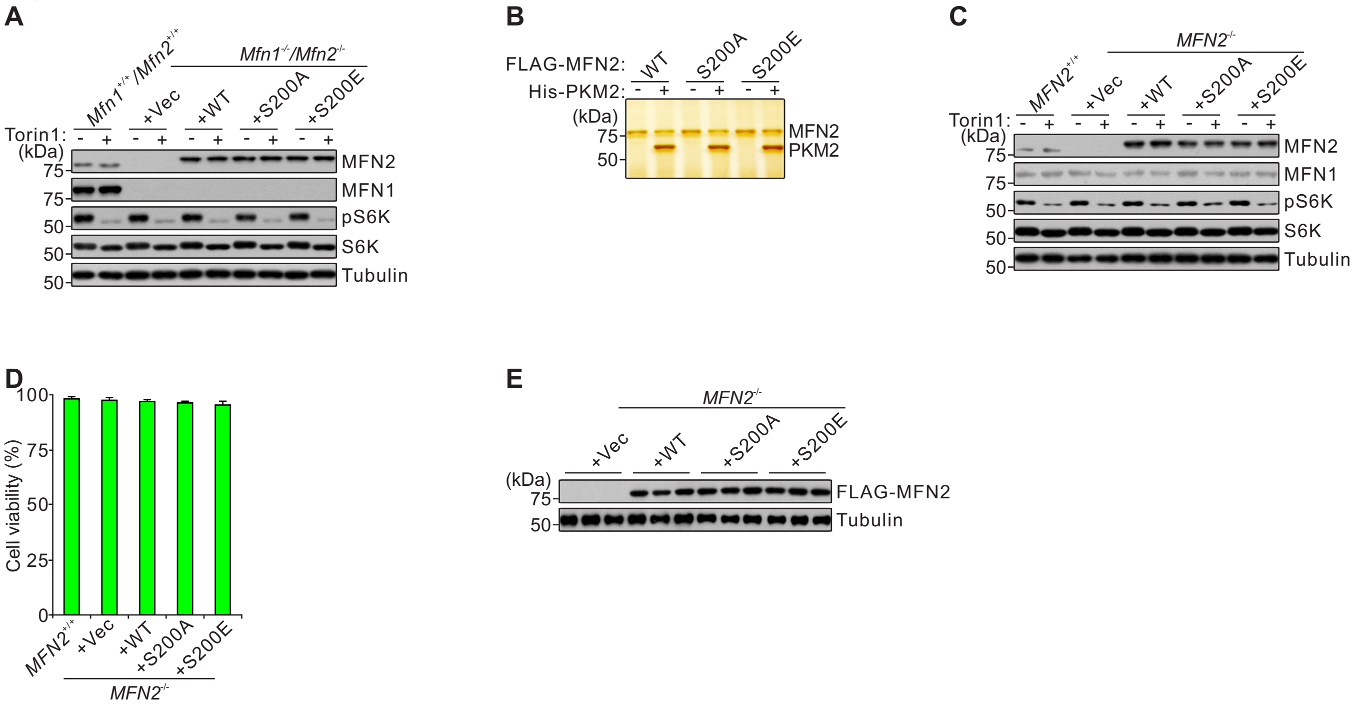

Figure S5

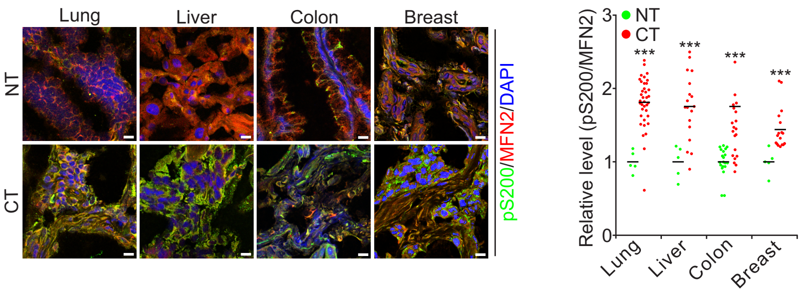

Supplement: Supplementary file 1 — Electronic supplementary material 1 (PDF 29044 kb) [file 13238_2019_618_MOESM1_ESM.pdf]
